# Furcation Involvement in Periodontal Disease: A Narrative Review

**DOI:** 10.7759/cureus.55924

**Published:** 2024-03-10

**Authors:** Syed Wali Peeran, Karthikeyan Ramalingam, Sathya Sethuraman, Madhumala Thiruneervannan

**Affiliations:** 1 Preventive Dental Sciences, Jazan University, Jazan, SAU; 2 Oral Pathology and Microbiology, Saveetha Dental College and Hospital, Saveetha Institute of Medical and Technical Sciences, Saveetha University, Chennai, IND; 3 Dentistry, Saveetha Dental College and Hospital, Saveetha Institute of Medical and Technical Sciences, Saveetha University, Chennai, IND; 4 Periodontics, Vinayaka Mission's Sankarachariyar Dental College, Salem, IND

**Keywords:** periodontal regeneration, pocket depth, periodontal pocket, factors, management, tooth survival, classification, therapy, periodontitis, furcation involvement

## Abstract

Furcation-involved teeth, commonly seen in dental practice, have a higher likelihood of needing extraction as the severity of periodontal furcation involvement increases. Studies consistently show that periodontitis in teeth with multiple roots significantly increases the risk of tooth loss, especially in the area where the furcation is involved. These furcation defects pose a major problem for dentists because of their location, accessibility issues, and the unpredictable healing process. The biggest hurdle in treating furcation defects is their irregular shape, which makes it hard to achieve complete debridement. While various treatments have been explored, non-surgical methods have not shown much success. This article comprehensively provides a review and discussion on the classification, assessment, and treatment options, including surgical and non-surgical management of furcation-involved molar teeth. Properly understanding the severity of the disease and its confounding factors and managing and treating the lesions appropriately have been shown to impart satisfactory survival rates for these teeth. Enhancing the understanding of managing these teeth can also lead to better outcomes for patients.

## Introduction and background

**Figure 1:**
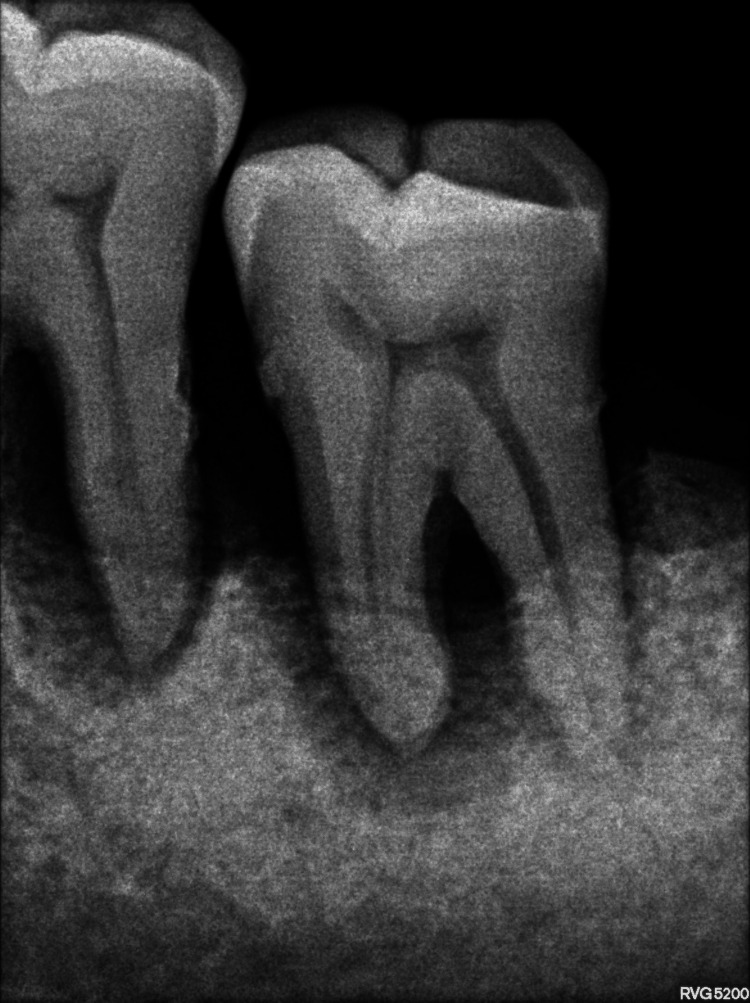
An intraoral periapical radiograph showing furcation involvement in mandibular molars Image credit: Dr. Karthikeyan Ramalingam, Dr. Jayanth Kumar

Furcation involvement (FI) refers to a condition in which the progression of the periodontal disease process invades the bifurcations and trifurcations of multirooted teeth. The resorption of bone and loss of attachment are the characteristics of FI (Figure 1) [1,2].

Etiology and predisposing factors

**Figure 2:**
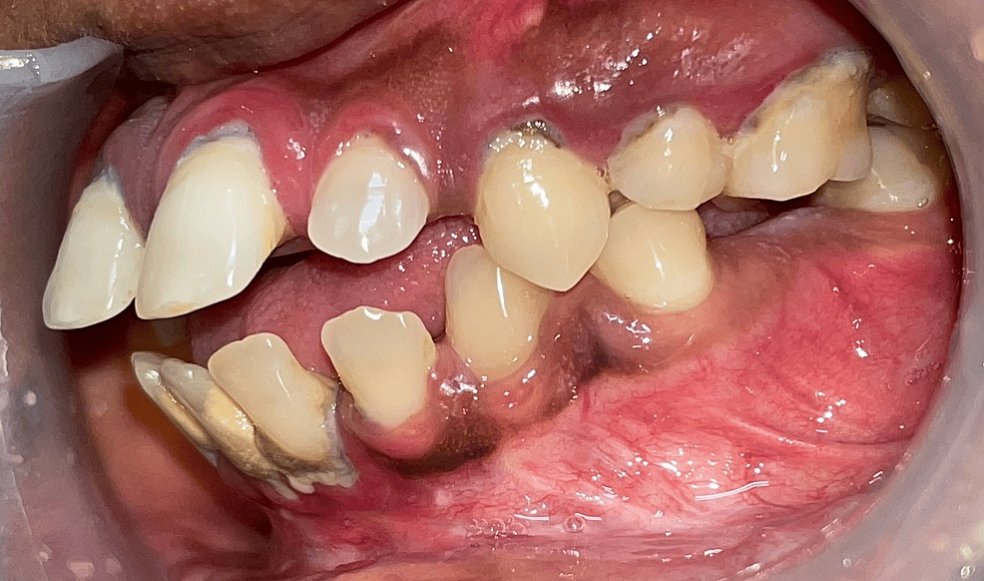
A clinical picture showing calculus deposits on teeth, generalized gingival inflammation and periodontal disease Image credit: Dr. Sankari, Dr. Karthikeyan Ramalingam

When the periodontal disease advances, it progresses and leads to the loss of the attached tissue involving the furcation area. This is the major etiological factor [1,2]. It is induced primarily by the persistence of the dental biofilm eliciting a host inflammatory response. Local anatomic factors could lead to the accumulation of dental biofilm and also hamper its removal (Figure 2).

Pulpal Periodontal Disease

Molars have been reported to have patent accessory canals in the furcation area. Endodontic infection can leach into the furcation area leading to isolated FI [2].

Trauma From Occlusion

It refers to tissue injury caused by distorted occlusion. Excessive abnormal occlusal forces can accelerate the destruction of the interradicular periodontal tissue in the presence of periodontal pathology. It could be acute or chronic and could be aggravated by local factors [2].

Iatrogenic Factors

Defective restorations approaching the furcation area can act as dental biofilm traps and lead to periodontal infection [2]. Endodontic perforations can also lead to communion and spread of endodontic infection into the furcation area leading to retrograde periodontitis and FI [3,4].

Root Fractures Involving Furcations

Vertical fractures that involve the root trunk can pass through the furcation. The bacterial flora from the oral cavity can seep into the pulpal and furcal tissues and infect them. The furcation and pulpal involvement in such cases is rapid, often has a poor prognosis and the affected tooth is generally extracted [2].

Local anatomic factors

Root Trunk Length

The root trunk's length dictates the treatment as well as the prognosis of the tooth involved. In teeth with short root trunks, the FI will be early, while in teeth with long root trunks, the FI will take a longer time and with much loss of attachment. Longer root trunks have a poor prognosis due to difficulty in scaling and root planing. Shorter root trunks are easier for periodontal therapy. Furcation-involved teeth with shorter trunks are more easily maintained post-treatment than those with longer root trunks [2,5,6]. The furcation on the first maxillary premolar opens mesiodistally and is at the apical third. In such cases, FI has a generally poor prognosis. Roots are generally fused in a third molar and have a very variable anatomy [7,8]. They seldom have periodontal FI and in the case of FI, they are extracted.

Cervical Enamel Projections

They are estimated to be present in 25% of mandibular teeth and 20% of maxillary teeth. According to the distance to the furcation entrance, Masters and Hoskins, in 1964, classified CEPs into three grades [2,9].

Enamel Pearl

Both cervical enamel projections and enamel pearls lead to the accumulation of plaque and isolated periodontal pocketing, which can permit severe periodontal infections and complicate scaling and root planing [2,9,10].

Interradicular Dimensions

The degree of separation is the angle of separation between the roots in multirooted teeth. A narrow furcation entrance hampers efforts to scale and root plane the area and hence would act as a reservoir of plaque and would adversely affect the prognosis. Teeth with widely separated roots can be easily cleaned and maintained and would be better candidates for treatment procedures such as hemisection [2,11].

## Review

Classification of furcation involvement

Pilloni and Rojas have summarized various classifications that have discussed furcation involvement in the literature and proposed their new classification [6]. Glickman in 1953 had described the extension and graded furcation defects from Grade I to Grade IV. Other systems are based on the amount of horizontal or vertical bone loss, anatomy of the furcation area, number of remaining bony walls, and morphology of the existing bone. The relationship between the root trunk and the horizontal or vertical bone loss has been classified [2]. However, there was no differentiation between clinically exposed and non-exposed furcation defects [6].

Diagnosis and prognosis

Furcation is clinically detectable only when attachment loss has occurred in the furcation area. Various diagnostic and prognostic indicators are available in the literature [2]. Detection and evaluation of the presence, type, location, and extent of FI are necessary to understand the prognosis and appropriately assign treatment options. The FI of both molars and premolars is diagnosed traditionally in clinics with ACE, ZA2, HO2 ZA3, Nabers, NS2, NP2C, and probes [11].

**Figure 3:**
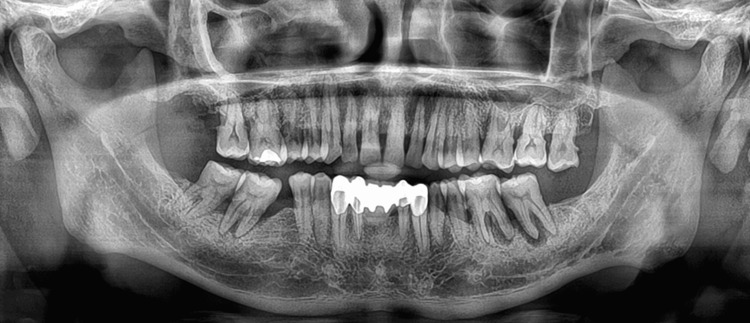
An orthopantomogram showing advanced periodontal disease with furcation involvement of right and left mandibular molars Image credit: Dr. Karthikeyan Ramalingam, Dr. Jayanth Kumar

Bone sounding of the anesthetized soft tissue of the furcation area helps plot the topography of the underlying alveolar bone. Radiographs such as intraoral periapical radiographs are also used for the diagnosis of FI. However, they are the least effective in detecting the furcation and its extent, especially in premolars and maxillary molars as the palatal roots superimpose and block the two-dimensional view and their precision in detecting initial FI is also low [12]. Clinical and radiographic assessment of FI teeth is mandatory as FI teeth if left untreated are at risk of progressive bone loss and tooth loss (Figure 3).

**Figure 4:**
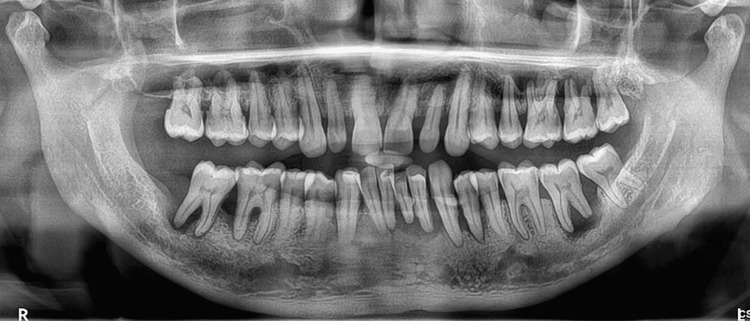
An orthopantomogram showing advanced periodontal disease with furcation involvement, periapical lesion and root resorption in mandibular molars Image credit: Dr. Karthikeyan Ramalingam, Dr. Jayanth Kumar

Computerized tomography, cone-beam computerized tomography, ultrasonography and new miniature periodontal endoscopes have also been used for diagnosing FI [13]. However, the use of these newer technologies is restricted due to their costs, lack of training and availability to clinicians. Furcation defects might lead to gradual loss of attachment, resorption of alveolar bone and even mobility of the tooth (Figure 4) [14].

FI affects the prognosis of the individual tooth. According to Nibali et al., the presence of FI increases the risk of tooth loss when it comes to molars. The chance of tooth loss is twofold more when furcation is involved [15].

Treatment modalities

**Figure 5:**
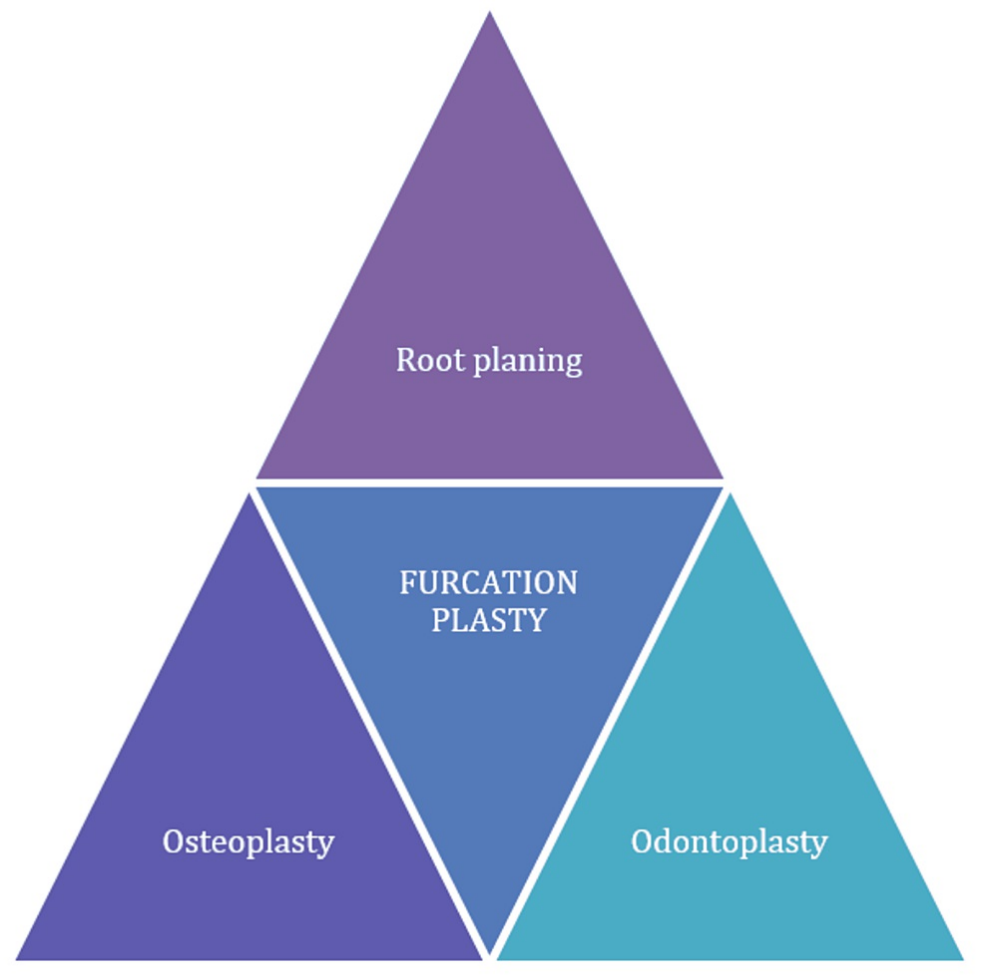
Components of furcation plasty Image credit: Syed Wali Peeran, Karthikeyan Ramalingam

The furcation area presents unique situations and challenges that limit the application of routine periodontal therapy. The special anatomy and morphology of the furcation area necessitate an array of special procedures and modified treatment approaches to overcome various confounding factors such as the size and shape of the furcation roof, entrance and the divergence of the roots along with the alveolar housing, and the varied nature and patterns of periodontal destruction [16]. Furcation plasty (osteoplasty, odontoplasty and root planing) is performed at the furcation level. This restores the architecture of soft tissues and makes cleaning easier (Figure 5).

**Figure 6:**
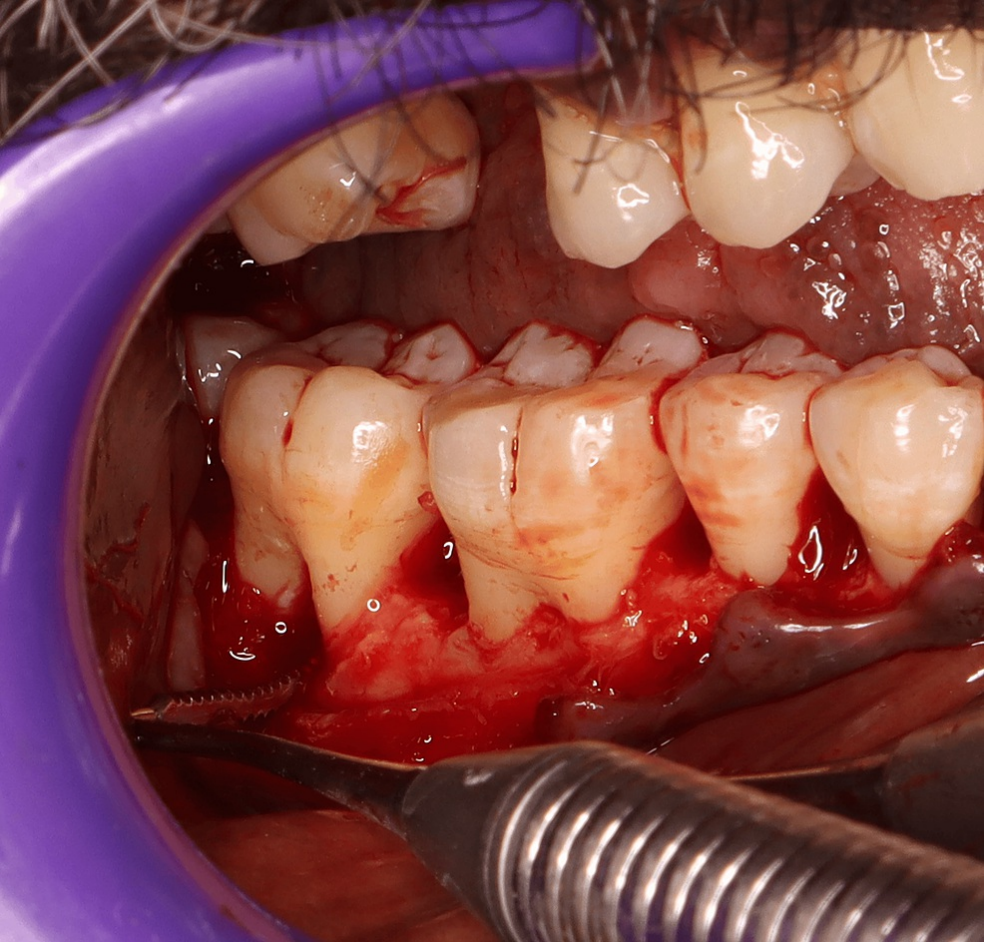
An intra-operative picture showing furcation plasty performed on mandibular molars Image credit: Dr. Sankari, Dr. Karthikeyan Ramalingam

A minor surgery is performed to create a tunnel in the furcation area, especially in the lower molar tooth. This helps to maintain oral health as the open furcation area enables easy access. It was a favored treatment procedure for Grade II and Grade III FI, especially for lower molars with long and divergent roots. The interradicular bone is removed and reshaped along the apical displacement of the soft tissue (Figure 6).

Postsurgically, soft tissue ingrowth is prevented with the placement of the periodontal pack material in the furcation region. The patient is responsible for the maintenance and cleaning of the tunnel with an interdental brush. Fluoride application is needed to prevent root caries. It is rarely performed today as complications such as dentinal hypersensitivity and root caries are common while post-surgical maintenance remains difficult [2].

Hemisection

The tooth is sectioned vertically through the crown and root [17]. Typically, hemisection is performed in the lower molars at the furcation level. This procedure is also known as bicuspidization or premolarization or segregation due to the transformation of a single molar into two teeth with single roots. If there is excessive bone loss, one half could be extracted and the other half could be restored as a premolar [2].

Root Amputation

Root amputation (of the mesio-buccal or distobuccal root) is a valuable procedure particularly applicable to three-rooted maxillary molars. It becomes more pivotal in cases where other treatment options are not possible [18].

Procedure: An inverse bevel incision is made. The buccal and palatal flaps are raised. Oral prophylaxis, scaling, and root planing of the molar are done and the granulation tissue is removed. Tapered diamond burs cooled with sterile water are utilized to sever the crown and root. An adequate space is maintained to facilitate the extraction of the root by root tip forceps. Diamond burs and stones are used after the root amputation to shape the remaining apical area of the crown and furcation region similar to the shape of a pontic so that maximal buccal access is available for maintaining oral hygiene. The periodontal pack is placed between the flap margins and the amputation site. Most root amputations involve the maxillary first and second molars. The factors to be considered when deciding which root to remove are as follows: the amount of supporting tissue around the roots, the root and root canal anatomy of the endodontic treatment, and the periapical condition [19].

**Figure 7:**
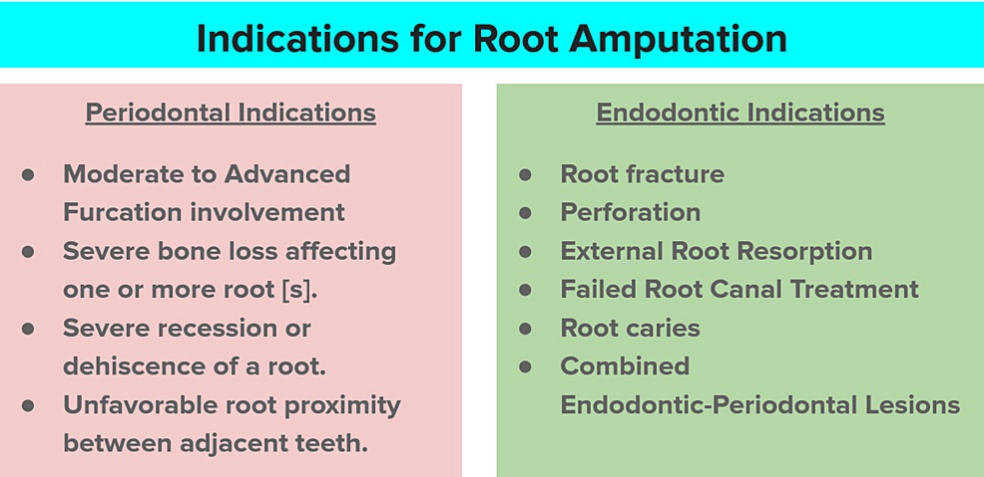
Indications for root amputation Image credit: Dr. Syed Wali Peeran, Dr. Karthikeyan Ramalingam

Root amputations or hemisections almost always result in irreversible pulpal damage; hence, root canal therapy (RCT) is done first. The coronal part of the tooth should be filled with mineral trioxide aggregate (MTA), which is a mixture of various calcium silicates. Sometimes the decision to perform root resection cannot be made until flaps have been reflected and the periodontal status has been surgically assessed. The RCT in such cases must be delayed until after the resection (Figure 7) [20].

Periodontal regenerative procedures

**Figure 8:**
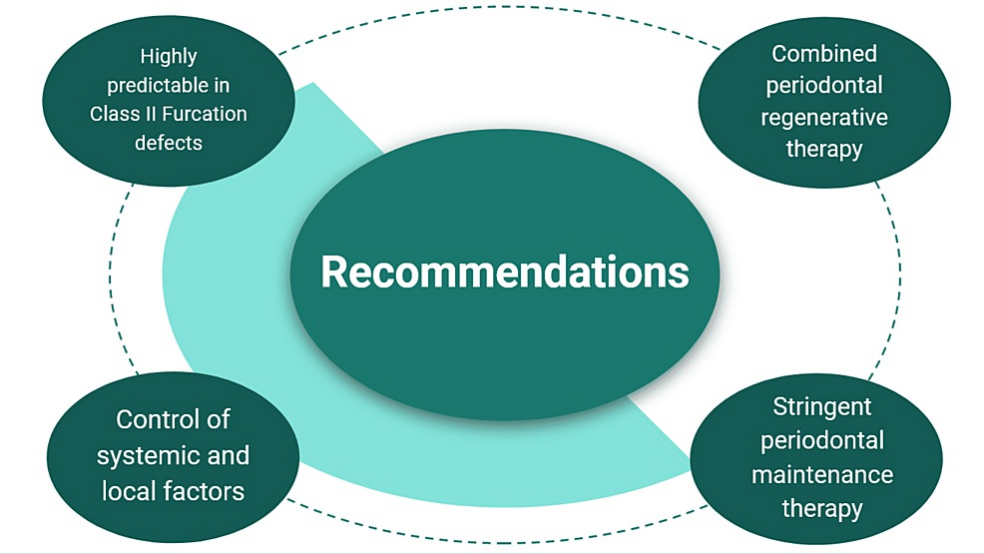
Recommendations for periodontal regenerative therapy in furcation involvement Image credit: Dr. Syed Wali Peeran, Dr. Karthikeyan Ramalingam

Regenerative periodontal surgical procedures are performed in cases where the interproximal bone is coronal to the defect, especially in class II furcation defects (Figure 8) [21,22].

Guided Tissue (Bone) Regeneration

**Figure 9:**
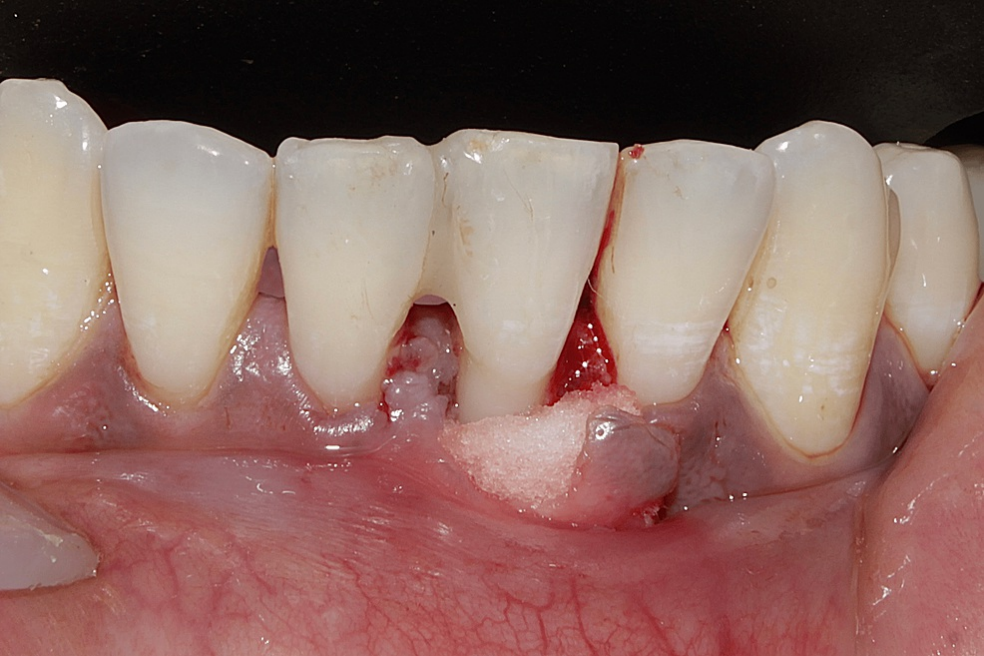
A clinical picture showing a guide membrane placed for periodontal regeneration Image credit: Dr. Sankari, Dr. Karthikeyan Ramalingam

Guided tissue regeneration (GTR) produces the most predictable results with class II furcation defects. GTR exhibits better clinical results when compared to the traditional flap surgery (Figure 9).

Growth Factors

**Figure 10:**
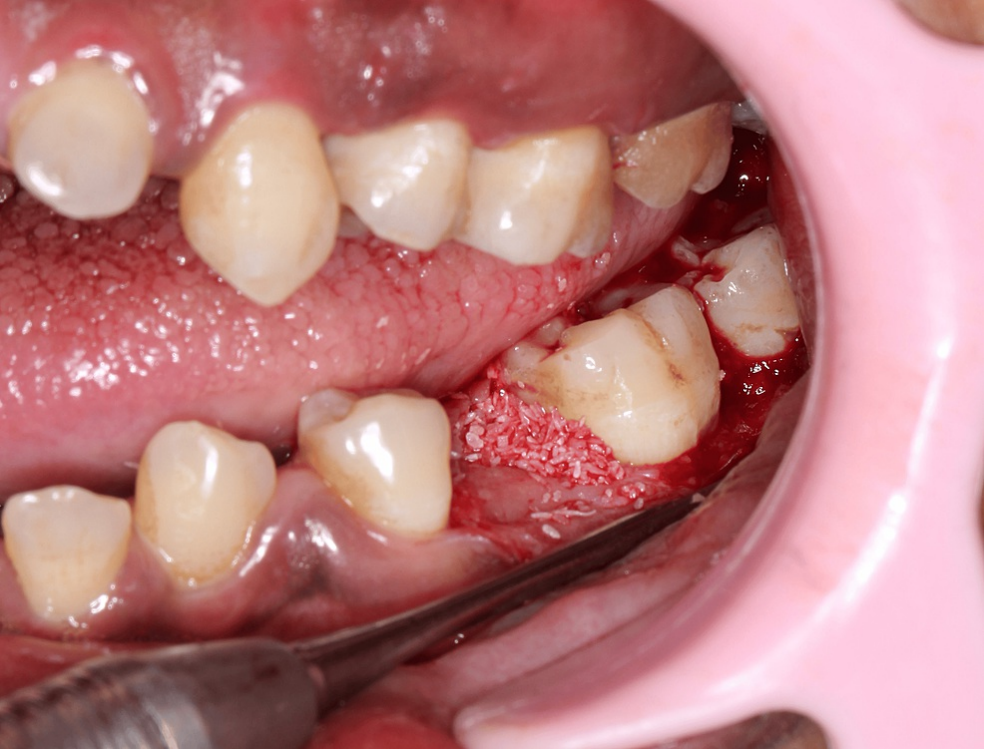
An intraoperative picture showing the bone graft placed in the mandibular molar region Image credit: Dr. Sankari, Dr. Karthikeyan Ramalingam

Autologous platelet concentrates, platelet-rich fibrin, and de-mineralized freeze-dried allograft (DFDBA) with recombinant human platelet-derived growth factor BB (rhPDGF-BB) may be beneficial as an adjunct to open flap debridement alone and bone grafting [22-24]. Enamel matrix derivative (EMD) is a sterile extract from the developing porcine fetal tooth material, which stimulates protein synthesis and mineral formation [24-26]. The use of EMD in furcations gives some additional reduction in horizontal furcation defect depth with resorbable GTR [27]. Platelet-rich plasma is an autologous concentration of thrombocytes. It is rich in growth factors. It can be easily procured from the patient and is inexpensive. It has been hypothesized to improve healing and help in handling bone grafts as it produces a sticky mass. Recombinant human bone morphogenetic protein-2 has shown some promising results in treating intrabony defects (Figure 10) [28].

Extraction

**Figure 11:**
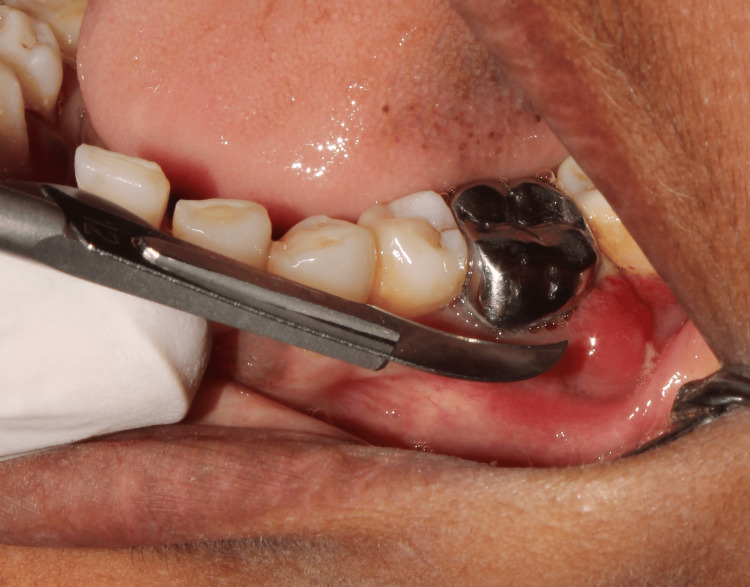
A clinical picture showing abscess formation in relation to the restored tooth Image credit: Dr. Vinod Krishna, Dr. Karthikeyan Ramalingam

Sometimes heroic efforts to save furcation-involved teeth are futile. The clinician must carefully evaluate the individual condition of the tooth as well as the overall prognosis of the case. Extensive caries, root caries, endodontic lesions, root resorption, and poor restorations can act as plaque traps and adversely affect the prognosis of teeth (Figure 11).

**Figure 12:**
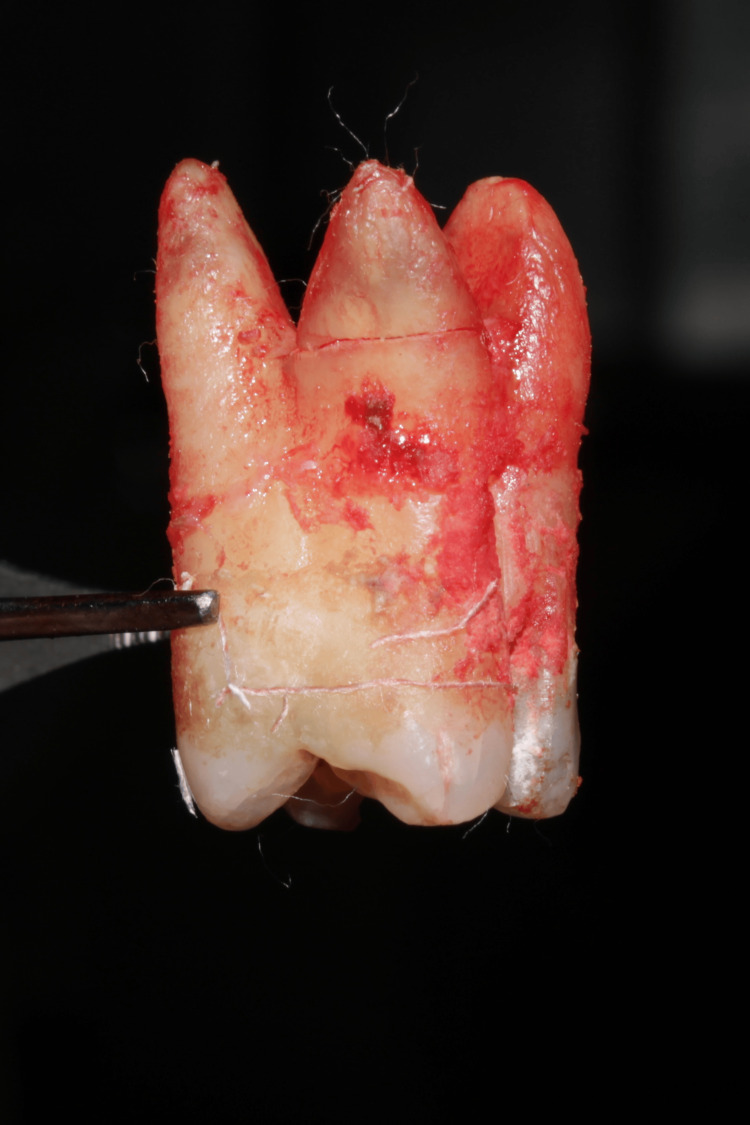
An extracted maxillary molar tooth Image credit: Dr. Murugesan, Dr. Karthikeyan Ramalingam

FI teeth that cannot be endodontically treated, those with extensive bone loss and severe mobility, and those that cannot be accommodated in the final therapeutic plan should be extracted (Figure 12) [2].

Systematic reviews have established the short‐term improvements shown by periodontal regenerative/reconstructive procedures compared to conventional surgical treatment in intrabony defects [28-32]. Jepsen et al. in their systematic review and meta-analysis of treatment for furcation defects reported that regenerative surgery has a superior outcome than open flap debridement [29]. Stavropoulous et al. have reported in their systematic review and meta-analysis that regenerative periodontal therapy have a better outcome in intrabony defects, in shallow pockets, and when administered as a combination therapy [30]. Jepsen et al. also reported that careful case selection with meticulous diagnosis, appropriate micro-surgical techniques, and high motivation in patients for periodic maintenance are crucial for a successful outcome [31].

## Conclusions

In conclusion, furcation involvement is a significant aspect of periodontal disease and adversely impacts the prognosis and treatment outcomes in the affected teeth. FI teeth require a careful assessment based on various degrees of severity. Comprehensive management and long-term prognosis are based on proper diagnosis and choice of treatment to ensure favorable treatment outcomes. The association of FI with systemic factors and its impact on tooth prognosis along with quality of life underscores the need for a comprehensive evaluation and targeted interventions in clinical dental practice.
